# Association Mapping of Flowering Time QTLs and Insight into Their Contributions to Rapeseed Growth Habits

**DOI:** 10.3389/fpls.2016.00338

**Published:** 2016-03-24

**Authors:** Nian Wang, Biyun Chen, Kun Xu, Guizhen Gao, Feng Li, Jiangwei Qiao, Guixin Yan, Jun Li, Hao Li, Xiaoming Wu

**Affiliations:** ^1^Key Laboratory of Biology and Genetic Improvement of Oil Crops, Ministry of Agriculture, Oil Crop Research Institute of the Chinese Academy of Agricultural SciencesWuhan, China; ^2^College of Horticulture and Forestry Sciences, Huazhong Agricultural UniversityWuhan, China

**Keywords:** growth habits, flowering time, GWAS, rapeseed, *B. napus*

## Abstract

Plants have developed sophisticated systems to adapt to local conditions during evolution, domestication and natural or artificial selection. The selective pressures of these different growing conditions have caused significant genomic divergence within species. The flowering time trait is the most crucial factor because it helps plants to maintain sustainable development. Controlling flowering at appropriate times can also prevent plants from suffering from adverse growth conditions, such as drought, winter hardness, and disease. Hence, discovering the genome-wide genetic mechanisms that influence flowering time variations and understanding their contributions to adaptation should be a central goal of plant genetics and genomics. A global core collection panel with 448 inbred rapeseed lines was first planted in four independent environments, and their flowering time traits were evaluated. We then performed a genome-wide association mapping of flowering times with a 60 K SNP array for this core collection. With quality control and filtration, 20,342 SNP markers were ultimately used for further analyses. In total, 312 SNPs showed marker-trait associations in all four environments, and they were based on a threshold *p*-value of 4.06 × 10^−4^; the 40 QTLs showed significant association with flowering time variations. To explore flowering time QTLs and genes related to growth habits in rapeseed, selection signals related to divergent habits were screened at the genome-wide level and 117 genomic regions were found. Comparing locations of flowering time QTLs and genes with these selection regions revealed that 20 flowering time QTLs and 224 flowering time genes overlapped with 24 and 81 selected regions, respectively. Based on this study, a number of marker-trait associations and candidate genes for flowering time variations in rapeseed were revealed. Moreover, we also showed that both flowering time QTLs and genes play important roles in rapeseed growth habits. These results will be applied to rapeseed breeding programs, and they will aid in our understanding of the relation between flowering time variations and growth habits in plants.

## Introduction

Plants have developed sophisticated systems to adapt to local conditions over the long history of evolution, domestication and natural or artificial selection (Anderson et al., 2011). These systems can be attributed to both physiological and genetic mechanisms, and they have resulted in divergent populations within a single species that grow in different geographic areas or different seasons. This finding further suggests that genomic regions have been targeted by selection and are therefore expected to exhibit characteristic changes in their levels and/or patterns of nucleotide diversity within species. Therefore, insight into these genomic targets and the dissection of the genetic variation of the related traits would improve our understanding of plant adaptation.

Of all the characters involved in growth adaptation, the transition from vegetative growth to flowering is the most crucial factor because it ensures that plants produce enough progeny for their sustainable development. Many plants synchronize their flowering to coincide with seasons by monitoring cues such as the temperature and photoperiod (Jung and Müller, 2009; Zuellig et al., 2014). Additionally, crops are adapted to different growth regions such as fall or spring sowing in different regions. These adaptation processes could enable plants or crops to form different growth habits, such as winter and spring types. For example, two types of annuals, namely biennials (winter annuals) and summer annuals were classified within *Arabidopsis thaliana* (Amasino, 2004). Usually, plants with different growth habits in one species show very different performance in terms of flowering times. Therefore, this finding suggests that the genetic factors that underlie flowering time variations play important roles in plant habits. However, although flowering time has been extensively studied, its contributions to growth habits are not yet understood. Hence, understanding the genome-wide genetic architecture that controls variations in flowering time and growth habits would be a central goal in plant genetics and genomics.

Rapeseed (*Brassica napus* L., 2*n* = 4*x* = 38; genome AACC) is one of the most important oilseed crops in the world. This plant is a member in the U triangle and close relations to the model plant *A. thaliana* (Nagaharu, 1935), the genetic control of flowering time and relevant genetic networks of which have been extensively studied. In *A. thaliana*, several pathways that control the flowering time have been revealed, namely the photoperiod, vernalization, gibberellic acid (GA), autonomous pathway, and thermal clock (Poethig, 2003). With comparative genetic analyses of these pathways between *A. thaliana, B. rapa* and *B. oleracea* with those of *B. napus*, many homologs of flowering time genes have been isolated, and they were shown to be associated with flowering time variations, such as five *FLC* homologs (*BnFLC1* to *BnFLC5*, and then nine were identified by another group), *BnFRI.A3* (*FRIGIDA*, designated *BnaA.FRI.a*) and three *BnFT* paralogs (*BnA2.FT, BnC6.FT.a* and *BnC6.FT.b*; Tadege et al., 2001; Wang et al., 2009, 2011; Zou et al., 2012). A number of studies also uncovered the genetic basis of flowering time variations in rapeseed and its relatives through linkage-based QTL mapping. Raman and co-authors reported flowering time as a complex trait in a doubled haploid (DH) rapeseed mapping population that is controlled by at least 20 loci, and it is localized on 10 different chromosomes. These loci each accounted for between 2.4 and 28.6% of the total genotypic variation for the first flowering and response to vernalization (Van Inghelandt et al., 2012; Raman et al., 2013). Another thorough study was also performed on the basis of a DH population and its derived reconstructed F_2_ population, which was planted in 11 field environments. Many significant-level QTLs (SL-QTL) and micro-real flowering time QTLs (MR-QTL) were detected in each environment (Long et al., 2007; Shi et al., 2009). In addition to these typical reports, there were also other studies that were aimed at dissecting the genetic architecture of flowering time variation in rapeseed and its relatives (Ferreira et al., 1995; Robert et al., 1998; Lou et al., 2007, 2011; Cai et al., 2008; Li et al., 2009; Wang et al., 2011; Hou et al., 2012; Uptmoor et al., 2012; Wu et al., 2012; Raman et al., 2013). However, all this research was usually performed with limited genomic information or small numbers of plant samples.

Genome-wide association mapping (GWAS, also known as GWA) was first used in human disease genetics, and it is primarily based on linkage disequilibrium (LD) in chromosomes (Rannala, 2001). Given that large numbers of plants with a wide genetic range and huge amounts of genomic information can be employed in GWAS, this type of study has attracted more and more attention within plant genomics and genetics (Bradbury et al., 2007; Glaubitz et al., 2014). For rapeseed, there were several case studies of some traits by GWAS of some traits. Cai et al. (2014) performed an association mapping of six yield-related traits in 192 inbred rapeseed lines with 674 molecular markers for genotype data (Cai et al., 2014). For the development of the genotyping technique, an SNP array with ~60 K SNP markers was produced by an international *Brassica* consortium. The genetic architecture of seed weights and seed quality was investigated through a genome-wide association study in a large rapeseed core collection panel with this SNP array (Li et al., 2014). Many known and novel significant marker-trait associations were discovered. In addition, another two typical studies were also performed using this 60 K SNP array in rapeseed. A diversity panel comprising 523 *B. napus* cultivars and inbred lines were genotyped with this SNP array and a genome-wide association study of flowering time was conducted. Totally, 41 SNPs distributed on 14 chromosomes were found to be associated with flowering time (Xu et al., 2016). Schiessl et al. (2015) identified 101 genome regions associating with the onset of flowering, 69 with plant height, 36 with seed yield and 68 cross-trait regions with potential adaptive value using 158 European winter-type *B. napus* inbred lines which were also genotyped using this 60 K SNP array (Schiessl et al., 2015).

In this study, we first performed a genome wide association mapping of flowering time by using the 60 K SNP array with a rapeseed core collection panel that contained 448 inbred lines. At least 40 QTLs showed significant association with trait variation. To understand the contributions of flowering time QTLs and genes to growth habits, selection signals corresponding to genomic regions that were related to divergent growth habits were screened in the rapeseed genome. A comparison of the flowering time QTLs and genes with these genomic regions revealed that both QTLs and genes play important roles in rapeseed growth habits. According to the performance shown by this study, we were able to provide a novel body of information for understanding the relationship between flowering time variations and growth habits in plants.

## Materials and methods

### Plant materials, growing conditions, and trait evaluation

A rapeseed core collection that was reported previously and included 448 inbred lines was used for plant material in this study (Li et al., 2014; Wang et al., 2014). All the pertinent information is listed in Table S1. More detailed information on these inbred lines can be queried through the Chinese Crop Germplasm Resources Information System (CGRIS: http://icgr.caas.net.cn/cgris_english.html). A request for accessions of interest can also be made through CGRIS or by communicating with the corresponding authors. The association panel was grown with 1–3 replications in the field in 2007, 2012 and 2013 (there was one replication in Wuhan in 2013, two replications in Yangluo in 2013 and three replications in Jiangxi at 2007 and 2012). Each accession was grown in a plot with five rows, and each row had 15 plants. The experiment was arranged as a randomized complete block design. In 2007 and 2012, the association panel was grown in the countryside of Jiangxi province (116.27 E, 28.37 N) and seeds were sown on October 1st and September 28th, respectively; in 2013, the association panel was grown in Wuhan (113.68 E, 30.58 N) and Yangluo (114.50 E, 30.38 N) in Hubei province and seeds were sown on Sep. 28th at both locations. All these places are situated along the Yangtze River, and they are classified as semi-winter growth environments for rapeseed (Wang et al., 2011; Hou et al., 2012; Cai et al., 2014). The agronomic practices were kept uniform in these four growth environments. The flowering times for the in field trials were recorded as the days from sowing to the date when the first flower had opened in 25% of the plants in each plot. To assign trait data for the four different growth environments, the flowering time corresponding to 2007 and 2012 in Jiangxi province and 2013 in Wuhan and Yangluo in Hubei province were referred to as JX2007, JX2012, WH2013, and YL2013, respectively.

### SNP development and genotyping

DNA that had been used for SNP genotyping was isolated from the young leaves of ~10 plants (pooled) for each of the 448 accessions (plant material was harvested from accessions grown in Wuhan 2013), according to a traditional large-scale CTAB method (Richards et al., 2001). Protocols for SNP genotyping, filtering and location were similar to those of (Li et al., 2014) and they were derived as part of a recently completed study (Wang et al., 2014). In brief, the Brassica 60 K Illumina® Infinium SNP array was used to obtain genotype data according to the manufacturer's protocols. SNP data were clustered and called up automatically with the Illumina BeadStudio genotyping software. Approximately 25,000 SNPs that did not show three clearly defined clusters that represent the three possible genotypes (AA, AB, and BB) were excluded. The probe sequences of each SNP were used to BLAST against *B. napus* genome sequence V4.1 (Chalhoub et al., 2014). The location of each SNP on 19 *B. napus* chromosomes was obtained by extracting the top hit positions of its corresponding probe sequence on the genome. Finally, SNPs that showed a minor allele frequency (MAF) of less than 0.05 were excluded from further analyses.

### Data analysis

Broad sense heritability was calculated as:

$$\begin{array}{l} H_{B}^{2}=\sigma_{g}^{2}\;∕\;\left(\sigma_{g}^{2}{+}^{\sigma_{g\text{e}}^{2}}{/}_{n}{+}^{\sigma^{2}}{/}_{nb}\right) \end{array}$$

where $\sigma_{g}^{2}$ is the genetic variance, $\sigma_{g\text{e}}^{2}$ is the interaction variance between genotypes and environments, σ^2^ is the error variance, n is the number of environments, and *b* is the number of replications in each experiment. This calculation is similar to Shi et al. (2015). Variance analysis was performed using “aov” function in R software and the formula was set as:

$$\begin{array}{l} \text{Aov}.\text{Fti}=\text{aov}\left(\text{Fti}\sim \text{Env}+\text{Gen}+\text{Envi}:\text{Gene},\text{data}=\text{FT}\right) \end{array}$$

“FT” indicates the imported data frame of original flowering time data, “Fti” indicates the total variance, “Env” indicates variance produced by environments, “Gen” indicates variance produced by genotypes and “Env:Gen” indicates interaction variance between environments and genotypes. In the variance analysis model, variations of genotypes, environments and interaction between genotypes and environments were considered as fixed effects.

Fixed effects in GWAS models were calculated with a Q or principal component (PCA) matrix, and random effects were calculated with a Kinship (K) matrix. SNPs with MAFs larger than 0.05 and with a distance to adjacent SNPs larger than the mean distance of all 20,342 SNPs were selected for Q and K matrix analyses. The Q matrix was predicted with the STRUCTURE v2.3.4 software package (Evanno et al., 2005). The number of groups/sub-populations (k) was set from 1 to 10 with the burn-in period, and the number of MCMC (Markov Chain Monte Carlo) replications after burn-in were both set to 100,000 under the “admixture mode.” Five independent runs were performed for each k number. The △k method described by Evanno et al. (2005) was used to determine the most likely number of groups/subpopulations. The PCA matrix was calculated by using all the SNPs that were identified by the GCTA tool (Yang et al., 2011), and the first five PCAs were used to construct the PCA matrix.

Six different models were employed for marker-trait associations in this study. In brief, these models were as follows: (1) the naïve-without control for fixed and random effects; (2) the Q model, which controls the fixed effect as the Q matrix; (3) the PCA model, which controls the fixed effects as the PCA matrix; (4) the K model, which controls the random effects as the K matrix; (5) the Q+K model, which controls fixed effects as the Q matrix and the random effects as the K matrix; and (6) the Q+K model, which controls the fixed effects as the PCA matrix and the random effects as the K matrix. The naïve, Q and PCA models were performed with the GLM algorithm, and the K, Q+K, and PCA+K models were run with the MLM algorithm implemented in the TASSEL 4.0 software (Glaubitz et al., 2014).

To identify true marker-trait associations, we applied two criteria to filter the signals produced by the selected association model. First, the *p*-values for all SNPs across all four environments were analyzed by Bonferroni multiple test to calculate the corresponding expected proportion of false discovery rates (FDR) with the “p.adjust” package in the R program (Benjamini and Hochberg, 1995). A true marker–trait association should show an FDR of less than 0.10. Second, a locus that is thought to have a marker-trait association should harbor at least two SNPs with *p*-values above the first criterion in a 1.5 Mb region or only one SNP showed marker-trait associations in at least two environments. We then identified candidate genes for the 40 flowering time QTL regions. For the identification of flowering time genes in rapeseed, we first collected all candidate flowering time genes in *A. thaliana* through published literature (Higgins et al., 2010); then the predicted protein sequences of rapeseed genome V4.1 were used as query to blast all *A. thaliana* flowering time genes, genes showed *E*-value below 1E-20 were considered as rapeseed flowering time genes. For each rapeseed flowering time gene, their nomenclatures and functions were predicted through their top hit in *A. thaliana.* For further analyses, flowering time QTLs were assigned names by adding the suffix “qFTi” (it represents the QTL of the flowering time), and their locations including the chromosome number and positions ranges together with “:” as conjunctions; e.g., qFTi:A01:4273503-5434402 represents the flowering time QTL that is located from 4273503 to 5434402 bp of chromosome A01.

To identify loci that contribute to rapeseed adaptation, an *F*_*ST*_ outlier detection method was used to detect the divergence between spring and winter rapeseed. This method was similar to that of a previous study with a slight improvement (Wang et al., 2014). In brief, an artificial selection between spring and winter rapeseed sub-populations was estimated on the basis of the pure drift model (Nicholson et al., 2002), by following the procedure as previously described. Genomic regions that were subjected to the above artificial selection were regarded as the primary factors that contributed to growth adaptation. The *F*_*ST*_ values were calculated for each SNP first, and then the average *F*_*ST*_ within a 0.5 Mb window with a 50 kb sliding bin for each of the 19 chromosomes was calculated. The mean *F*_*ST*_ was set at zero when the 0.5 Mb windows contained fewer than 2 SNP markers. Subsequently, the genomic regions (0.5 Mb windows) showing with *F*_*ST*_ value above the 10th percentile were regarded as the targets of artificial selection. For the classification of the growth types, the types were carefully collated and curated on the basis of their place of origin, recorded pedigrees, breeding records and previous field performance.

## Results

### Flowering time variations in a rapeseed core collection panel

**Figure 1:**
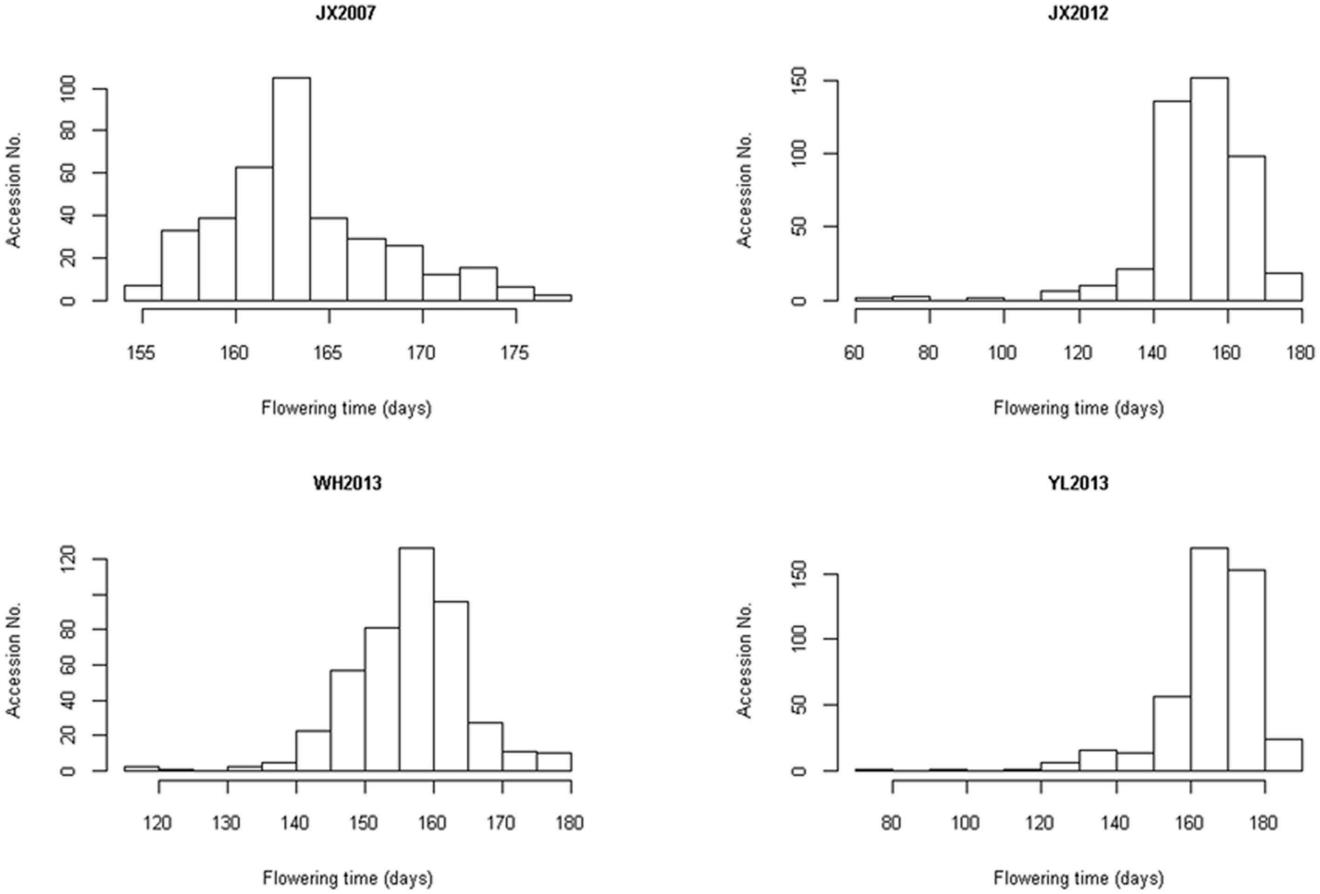
**Distribution of flowering time in four different environments**. The rapeseed core collection panel of 448 lines was planted in four different environments for 3 years, namely 2007, 2012, and 2013. In 2007 and 2012, the panel was grown in Jiangxi, and the codes for these two environments are JX2007 and JX2012, respectively. In 2013, the panel was grown in Wuhan and Yangluo, and the codes for these two environments are WH2013 and YL2013, respectively. The X axis indicates the flowering time (days) and the Y axis indicates accession number.

**Figure 2:**
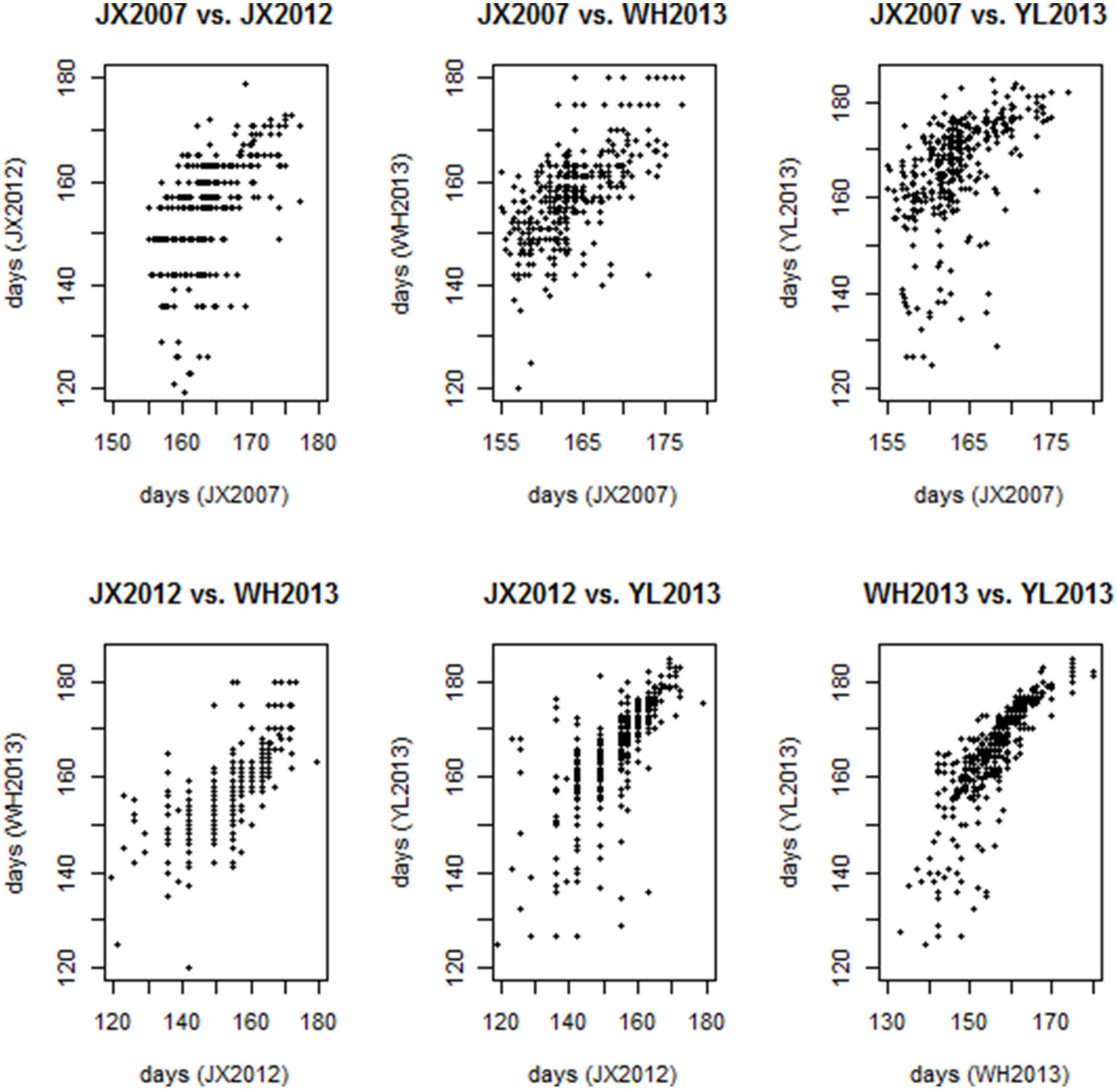
**Pair-plots of flowering time correlation coefficients**.

According to the trait evaluation that was performed in the fields, extensive flowering time variation was observed between or within environments (Table 1 and Figure 1). Figure 1 and Table 1 showed flowering times ranging from 155 to 177 days in environment JX2007, 77 to 182 days in environment JX2012, 75 to 181 days in environment YL2013 and 115 to 164 days in environment WH2013. In comparing the flowering times across environments, although the correlation between pairs of environments showed in a close level, large variations were also clearly observed (Table 2 and Figure 2). YL2013 showed the longest average flowering time at 164.8 ± 12.8 days, whereas WH2013 showed the shortest with 154.6 ± 8.6 days (Table 1). These data indicate that the flowering time for rapeseed could be greatly affected by environment. To test the effects of genotypes, environments and their interactions on flowering time variation, a variance analysis was conducted on our core collection panel. Table 1 reveals that the genotype (G), environment (E) and genotype-by-environment interaction (G × E) have significant effects on flowering time variation (*p* < 0.01). According to this calculation, the broad sense heritability of the flowering time is ~83% (Table 1).

**Table 1:** **Analysis of variance for rapeseed flowering time**.

**Flowering time data**					
	**Mean**	***SD***	**CV (%)**	**Min**.	**Max**.
JX2007	163.4	4.5	2.8	155.0	177.0
JX2012	159.8	12.9	8.1	77.0	182.0
WH2013	154.6	8.6	5.6	115.0	164.0
YL2013	164.8	12.8	7.8	75.0	181.0
**Variance analysis**					
**Source**	**DF**	***SS***	**MS**	***F*****-value**	**Pr(**>**F)**
G (genotype)	447	241,128	539	44	<2E-16
E (environment)	3	106,783	35594	2900	<2E-16
G × E	1172	126,352	108	8.8	<2E-16
Residuals	2062	25,306	12.3		

SD indicates the standard deviation of the flowering time. CV indicates the coefficient of variation. The unit for the mean, SD, Min. and Max. is days. DF indicates the degrees of freedom, SS indicates the sum of squares, MS indicates the mean square.

**Table 2:** **Flowering time correlation coefficients between pairs of environments**.

	**JX2012**	**WH2013**	**YL2013**
JX2007	0.428^***^	0.631^***^	0.526^***^
JX2012		0.702^***^	0.730^***^
WH2013			0.850^***^

*** indicates a p-value of less than 0.001.

### Evaluation of association mapping models

**Figure 3:**
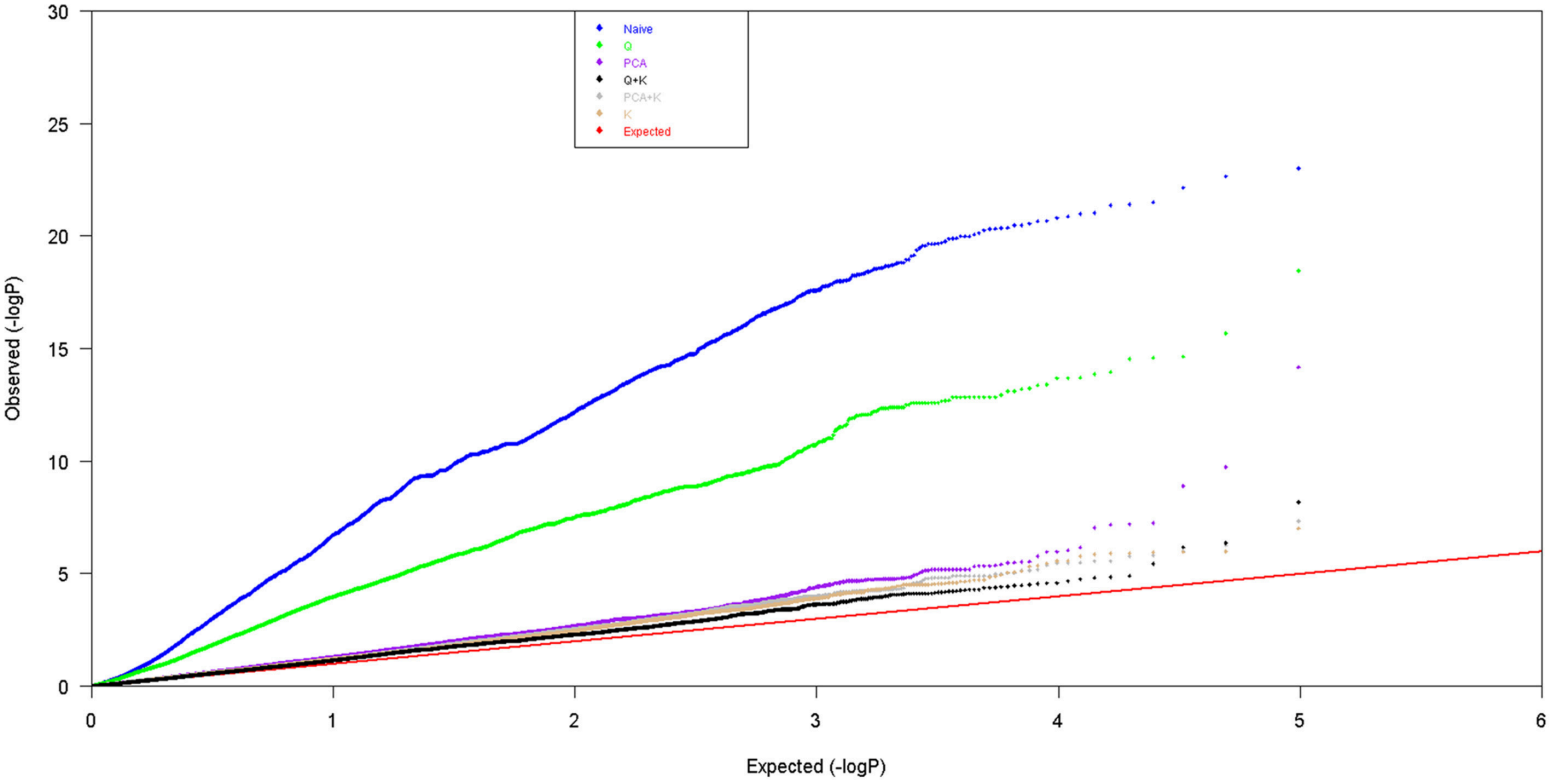
**QQ plots of the estimated −log_10_ (*p*-value) from association analyses using six different models**.

To create a Q matrix for all 448 rapeseed accessions, the Δk method was used to determine the most likely number of groups/sub-populations. The results showed that *k* = 2 produced the highest Δk value, and therefore, it was selected to create the Q matrix, This result was similar to that of (Wang et al., 2014). To assess the utility of each GWAS model, observed *p*-values [observed −log_10_ (*p*-value)] across all four environments from each model and their expected ranked values [expected −log_10_ (*p*-value)] were plotted in a quantile-quantile (QQ) figure (Figure 3). An ideal model should show uniformity between the observed and expected *p*-values in the QQ plot. According to Figure 3, the Q+K, PCA+K, K and PCA models showed much better correlation than naïve and Q models. Slight differences were observed among the Q+K, PCA+K, K and PCA models. However, the Q+K model was somewhat better than the others, therefore, this model was selected to detect marker-trait associations.

### Genome-wide association studies and candidate gene identification

**Figure 4:**
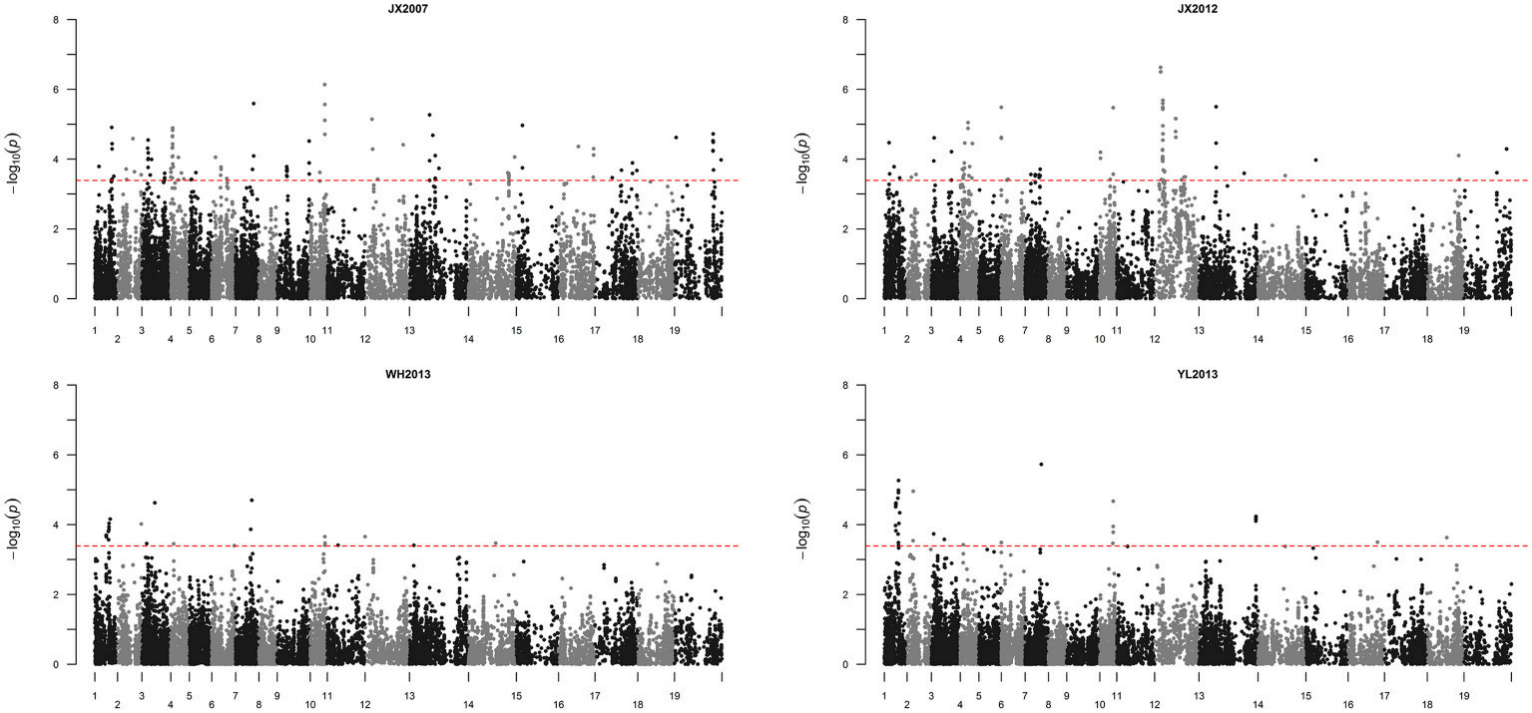
**Manhattan plots of association analysis using the Q+K model for rapeseed flowering times in four environments**. The X-axis indicates the SNP positions on 19 chromosomes within the rapeseed genome. They were arranged from A01 to A10 and C01 to C09 with alternating color plots of black and gray. Numbers 1 to 10 correspond to chromosomes A01 to A10, and numbers 11 to 19 correspond to chromosomes C01 to C09. The red dashed line in each environment indicates that the threshold *p*-value is equal to 3.83 × 10^−4^. The X-axis indicates the −log_10_ (*p*-value) for each SNP.

By using Bonferroni multiple testing to control the false discovery rate in the Q+K association model (Benjamini and Hochberg, 1995), a *p*-value of 4.06 × 10^−4^ [−log_10_ (*p*-value) = 3.39] revealed an FDR of 0.10. This criterion is usually selected as the threshold in GWAS studies (Cai et al., 2014). Therefore, SNPs with *p*-values of less than 4.06 × 10^−4^ were considered candidates for flowering marker–trait association. In total, 312 SNPs showed marker–trait associations in all four environments based on the threshold. Because Wang et al. reported that the distance between SNPs approached 1.5 Mb, the average *r*^2^ was 0.1 (Wang et al., 2014). This LD level usually indicates that there is nearly no linkage between the markers; thus, we defined SNPs within a 1.5 Mb window as a single locus. The 312 pairs of marker–trait association SNPs could be merged into 40 loci (Table S2 and Figure 4). Twenty-two SNPs could not be assigned into any marker–trait association loci because they did not meet the criterion of at least two SNPs with *p*-values above the threshold in a 1.5 Mb region. According to these data, we can conclude that we have identified 40 flowering time QTLs by genome-wide association mapping in the rapeseed core collection from four environments.

Of these 40 flowering time QTLs, 19 could be detected in at least two environments and they are located on chromosomes A01, A02, A03, A04, A06, A07, A10, C02, C03, C04, and C09 (Figure 4 and Table 3). Four QTLs, namely qFTi:A04:257040-4734286, qFTi:A07:14463578-18554138, qFTi:A10:13375104-15191366 and qFTi:C02:6956919-13653054 could be detected in all four environments. Two could be detected in three environments, namely qFTi:A01:9984823-11863045 and qFTi:A01:13863759-15685448. qFTi:C02:6956919-13653054 showed the highest probability of marker–trait association with −log_10_ (*p*-values) equal to 6.5, and it showed the largest contribution rate to flowering time variance with *R*^2^ equal to ~8.77%.

**Table 3:** **Marker–trait associations were detected in at least two environments for rapeseed flowering times**.

**Chr**.	**−log10 (P)^a^**	**Max R2 (%)**	**Range (bp)^b^**		**Environments^c^**
A01	4.47	4.73	4,273,503	5,434,402	JX2007, JX2012
A01	4.62	4.89	9,984,823	11,863,045	JX2012, WH2013, YL2013
A01	4.92	5.21	13,863,759	15,685,448	JX2012, WH2013, YL2013
A02	3.72	4.56	8,776,742	9,248,051	JX2007, JX2012
A02	3.55	4.33	24,150,742	24,237,453	JX2007, WH2013
A03	4.61	4.88	2,419,855	2,877,343	JX2012, YL2013
A03	4.55	5.57	5,046,910	6,515,058	JX2007, WH2013
A03	4.63	4.86	13,297,841	13,297,841	WH2013, YL2013
A04	4.89	6.00	257,040	4,734,286	JX2007, JX2012, WH2013,YL2013
A04	5.05	5.81	7,743,947	10,942,653	JX2007, JX2012
A04	4.45	4.70	11,898,475	13,460,703	JX2007, JX2012
A06	4.62	4.93	42,625	73,593	JX2012, YL2013
A07	5.59	6.91	14,463,578	18,554,138	JX2007, JX2012, WH2013, YL2013
A10	3.62	4.41	9,835,903	10,695,100	JX2007,JX2012
A10	6.13	7.87	13,375,104	15,191,366	JX2007, JX2012, WH2013, YL2013
C02	6.50	8.77	6,956,919	13,653,054	JX2007, JX2012, WH2013, YL2013
C03	5.27	6.53	17,612,972	20,733,429	JX2007, HX2012
C04	3.53	3.71	27,699,177	27,699,177	JX2012, WH2013
C09	4.72	5.79	39,312,343	43,429,210	JX2007, JX2012

a −logP is the −log_10_ (p-value) for the lead SNPs.

b The locations of SNPs show the −logP above the criterion of 3.42.

c Codes are identical with those of Figure 1.

Flowering time genes in *A. thaliana* were obtained through published literature (Higgins et al., 2010). Genes that were involved in flowering time pathways in rapeseed were obtained according to close relation between *A. thaliana* and the *Brassica* genus (see Materials and Methods).Totally, 1520 candidate flowering time genes were identified in the newly released *B. napus* genome. All candidate flowering time genes and their corresponding locations on rapeseed chromosomes are listed in Table S3. By comparing the locations of flowering time QTLs with flowering time candidate genes on rapeseed chromosomes, we were able to identify candidate genes for 25 of the 40 flowering time QTLs (Table S2). For these 25 QTLs, 19 showed more than one candidate gene in a locus. For example, there are 12 flowering time genes located within qFTi:A04:257040-4734286; they are *CDF3, AGL24, CKA2, AGL24, CIB1, AGL18, PIE1, AREB3, CDF2, AGL32, CKA2*, and *ARP6*.

In the QTL regions with candidate genes, we found that some were similar to those identified in previous studies. Two candidate genes within qFTi:A03:5046910-6515058, namely *FRI* and *FLC*, were also reported by two previous studies (Wang et al., 2011; Zou et al., 2012). In using an important flowering time gene called, *FLC*, as a common anchored marker, we found that qFTi:A10:13375104-15191366 was also reported by previous flowering time QTL mapping using a DH population (Long et al., 2007; Wang et al., 2011; Hou et al., 2012; Zou et al., 2012). The *FLC* gene in this locus that is involved in the vernalization pathway is located at 14,998,617 to 15,003,197 bp; it is only ~145 kb from the lead SNP (SNPs showed the largest *p*-value and *r*^2^ for this QTL), Bn-A10-p14914898 (Tables S2, S3). Additionally, as information is obtained, some flowering time QTLs would be novel, such as qFTi:A01:4273503-5434402 and qFTi:A04:257040-4734286. According to these results, we can conclude that a number of loci and genes explain flowering time variations in the core collection.

### Contributions of flowering time QTLs and genes to rapeseed growth habits

**Figure 5:**
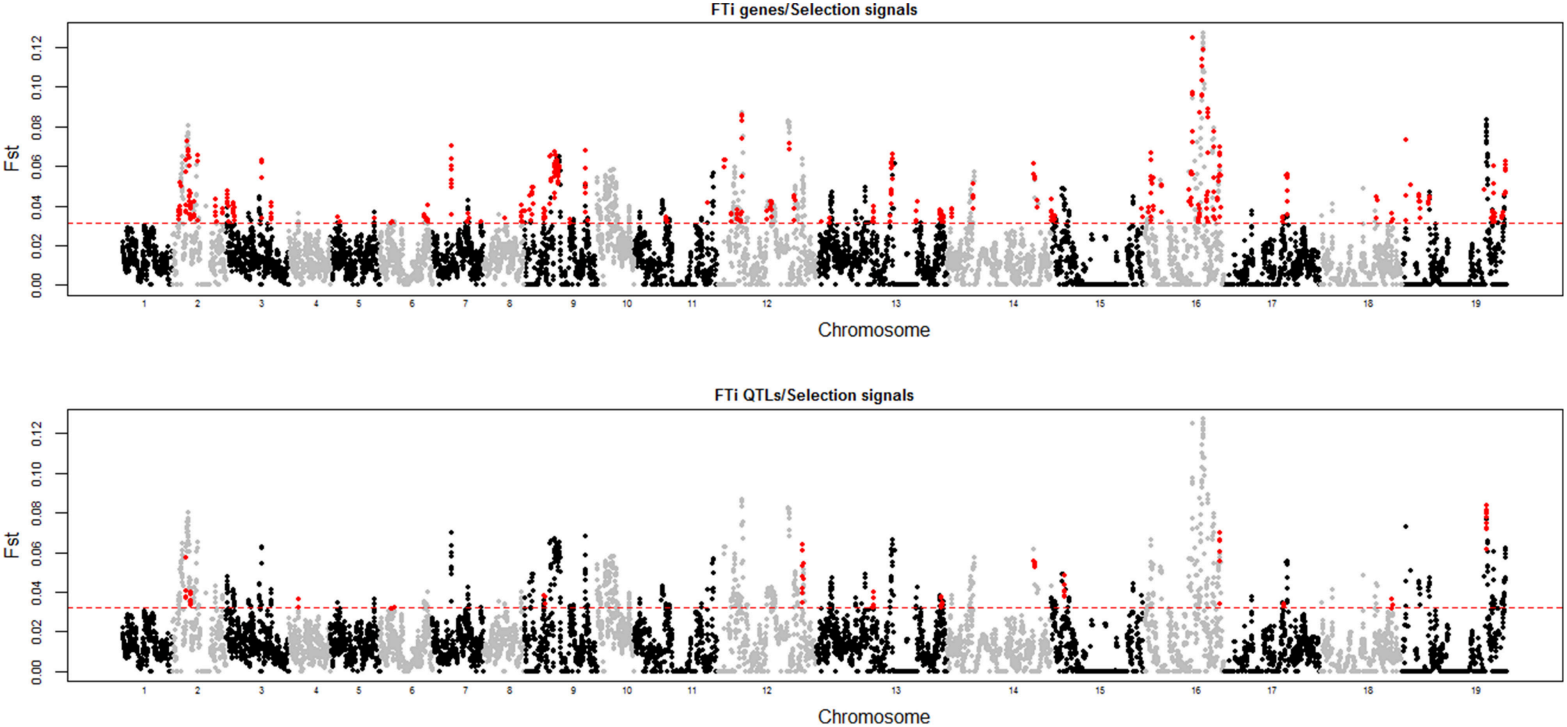
**Comparisons of selection regions with locations of flowering time genes (up) and QTLs (bottom)**. The X-axis is identical to that of Figure 4. The Y-axis indicates the *F*_*ST*_ values between the spring and winter rapeseed sub-populations. The red dashed lines indicate the threshold of the 10th percentile of the *F*_*ST*_-window. The red plots indicate the flowering time genes (up) or QTLs (bottom) in these locations and the *F*_*ST*_ values in these locations are also above the 10th percentile.

To investigate the contributions of flowering time QTLs and genes to rapeseed growth habits, we first identified the influence of artificial selection in the formation of different growth types in rapeseed. The growth type for each accession in our core collection panel is listed in Table S1. Because it was difficult to determine the exact growth type characteristics for semi-winter rapeseed, this growth type was excluded from the analysis. There were 106 spring and 153 winter rapeseed growth types (Table S1). Because *F*_*ST*_ is not strongly affected by ascertainment bias, it is better suited for analyzing data that are generated by SNP chips (Albrechtsen et al., 2010). We performed a genome-wide scan for selection signatures in our set of 259 *B. napus* accessions including spring and winter rapeseed growth types by estimating Wright's *F*_*ST*_ with a sliding window method (see Materials and Methods), and the 10th percentile *F*_*ST*_-window was said to include loci that were subjected to artificial selection, which was responsible for the divergence of rapeseed adaptation. A total of 117 loci equal to 124.0 Mb of the genome met our criterion. This finding indicated that nearly one-tenth of the genome (the total size for the whole rapeseed genome is approximately 1.2 Gb) contributed to the divergence that occurred between spring and winter growth habits for rapeseed. These 117 loci were simply named “selection regions.” We then compared these 117 regions on 19 chromosomes with all 40 flowering time loci. Twenty flowering time QTLs overlapped with 24 selection regions (Figure 5 and Table S4). Most of the 20 QTLs made large contributions to the flowering time variation and could be detected in more than one environment in an association mapping study (Table 3 and Table S2). When comparing the locations of flowering time candidate genes in the rapeseed genome within these selection regions, 224 out of 1520 flowering time genes fell into these 81 selection regions (Figure 5 and Table S4). Based on an investigation of annotation information for these 224 genes, most are involved in flowering time regulation through pathways for the photoperiod, vernalization, GA, autonomous pathway, and thermal clock (Table S3).

## Discussion

In this study, we were able to detect 40 flowering time QTLs by genome-wide association analysis by using a large rapeseed core collection panel. However, we did not know which of these QTLs had already been reported and which were new. Therefore, comparisons of our results with previous studies by anchoring a known marker sequence to rapeseed chromosomes would answer this question. Because the information from many for lots of studies was not complete, we only compared our results with TN DH mapping population and another two similar studies which also used 60 K SNP array to perform genotyping. Totally, we found that 7 of the 40 flowering QTLs in this study have been reported by studies on TN DH mapping population, 5 have been reported by studies performed on 158 European winter-type *B. napus* inbred lines (Schiessl et al., 2015) and 5 have been reported by studies performed on a diversity panel comprising 523 *B. napus* cultivars and inbred lines (Xu et al., 2016) (Table S2). In sum, 13 of the 40 flowering QTLs in this study can be found in at least one of other studies which were used in comparison. Given the limited information and different algorithms between linkage (QTL mapping) and linkage disequilibrium mapping (GWAS), we speculated that the numbers of QTLs reported by previous studies would be larger than 13. Of these 13 common QTLs, some of them were reported in many environments, such as qFTi:A10:13375104-15191366 and qFTi:C02:6956919-13653054, which indicated that these loci could control the flowering times under different growth conditions, and they were very stable. For further insights into these novel QTLs in Table S2, they tended to have no candidate genes. This finding suggested that further studies should focus on these QTLs and it also revealed the powerful nature of the GWAS when used with our core collection panel. Moreover, in the association mapping analysis, some QTLs showed very high contributions to flowering time variations, such as qFTi:C02:6956919-13653054, which explained ~9% of the variance (Table 3 and Table S2). Because the SNP markers used in this study are also publicly available in the Brassica Consortium, the information on marker-trait associations and candidate flowering time genes can guide marker assistant selection (MAS) in rapeseed breeding programs to come. Therefore, based on all the results from this study, we can conclude that we have provided very valuable data and resources for further rapeseed research.

In this study, we evaluated the flowering time data from four independent environments over three different years. Large variations across different environments were shown in Figure 1, and thus, this panel should be highly suitable for a GWAS study. Moreover, strong interactions between genotypes and environments were also revealed. This finding indicates that variations in environments make large contributions to flowering time variations. In addition, when comparing our results with previous flowering time studies for rapeseed, many of the flowering QTLs reported by others were not detected in this study. For example, QTLs on linkage groups C09 (Luo et al., 2014) and A05 (Long et al., 2007) could not be identified in the present study. This omission may be attributed to the fact that we only grew the rapeseed core collection panel in four environments: JX2007, JX2012, WH2013, and YL2013. Though they could be assigned to different places or different growing years, all of them were still located in central China along the Yangtze River. Therefore, the diversity of climate factors, such as the temperature, photoperiod and humidity, within these four environments is not sufficient to uncover all the mechanisms underlying the flowering time variation. This finding suggests that we should grow this collection panel in more environments to uncover more QTLs that are associated with the flowering time variation.

During further analysis of the flowering time QTL regions and their candidate genes (Table S2), it was common to find more than one candidate flowering time gene within a single QTL. This result suggests some possible patterns in the rapeseed genome and in flowering time regulation. First, genes that perform functions in the same or related pathways tend to be located in clusters in the genome. Similar discoveries were also reported in a number of other studies (e.g., Lee and Sonnhammer, 2003; Lee and Chang, 2013). This phenomenon would be more likely because rapeseed is an allotetraploid plant, and the two progenitors, that is, *B. rapa* and *B. oleracea*, also have a close genetic relationship. Second, different flowering time genes that are clustered in the same genomic region in rapeseed would have experienced co-evolution or co-selection. We found divergent signals from *F*_*ST*_ between spring and winter rapeseed types. The genomic regions with outlier *F*_*ST*_ values were usually the targets of natural or artificial selection. Genetic factors including coding and non-coding elements within those targets in the same sub-population should have undergone similar selective pressures. Therefore, these elements should show the same patterns of evolution or selection. This assumption is very similar to a previous study on flowering time genes. Zou et al. noted that functionally related flowering time genes are tightly linked in Brassica species, and these tightly linked flowering time genes exhibited co-expression patterns (Zou et al., 2012). However, the mechanism for underlying the flowering time gene clustering in the rapeseed genome is still not clear, and more studies should be performed on this subject.

According to the investigation of genomic regions that corresponded to growth habits in rapeseed, 117 loci equal to 124 Mb of the genome showed possible selection signals (Table S4). Given that the total size of genome is ~1.2 Gb, this finding suggests that 10% of the genome would be the target of selective pressures during the growth habit-shaping processes in rapeseed. There is only a ~7500–10,000 year history for this plant and as a result of slow linkage disequilibrium decay in the genome (Nagaharu, 1935; Qian et al., 2014; Wang et al., 2014), many genetic elements located in the selection regions would have no direct effects on growth adaptation but would only be detected by “linkage-drag.” For insight into the procedures used to screen of the selection regions in this study, a low stringent threshold for an *F*_*ST*_ above the 10th percentile value was employed. This threshold would overestimate the selection signals in the whole genome. However, because the transition from vegetative growth to flowering is one of the crucial direct factors in determining plant growth habits (Anderson et al., 2011), combining the analyses of flowering time QTLs and candidate genes would have high efficiency and accuracy at uncovering the mechanisms that underlie growth adaptation. This result also suggests that the result of QTL or candidate genes findings that were involved in growth habits in this study would be very reliable. Additionally, we found that 20 out of 40 flowering time QTLs overlapped the 24 selected loci that are important in determining rapeseed growth habits (Figure 4 and Table S4). This finding indicates that ~50% QTLs could be involved in this biological process. By contrast, 224 out of 1520 flowering time candidate genes were detected when determining rapeseed growth habits (Figure 4 and Table S4), which is ~15% of the total flowering time genes. This pattern may indicate that although there are numerous flowering time genes in the rapeseed genome, not all genes perform their full functions. This lack of full activity is very common for duplicated genes in complex genomes (Wang et al., 2013). Additionally, although large flowering time variations were observed among the four environments for our 448 inbred lines, these environments for GWAS still belong to the same climate area (semi-winter) along the Yangtze River in central China, and as a result, not all QTLs that were responsible for flowering time variation in our panel were identified. More overlaps would be observed between the selection and flowering time QTLs if the GWAS studies were performed in more divergent environments.

In this study, a wide distribution and large variation of flowering times were observed in the rapeseed core collection. Based on a variance analysis of flowering time data, the wide inheritance of this trait was also observed. Moreover, both the environment and the interactions between environments and genotypes showed significant effects in controlling the flowering time variation. Following genome-wide association mapping, 312 SNPs showed marker–trait associations in all four environments based on the *p*-value threshold of 4.06 × 10^−4^ and 40 QTLs showed a significant association with flowering time variation. These SNPs are distributed on 17 of the 19 chromosomes, with the exceptions of chromosome A08 and C01. These marker–trait associations and flowering time genes can be easily applied to advance rapeseed breeding programs. To further understand the contributions of flowering time QTLs and genes to growth habits, selection signals related to divergent adaptation in rapeseed were scanned at a genome-wide level. A locations comparison of flowering time QTLs and genes with these genomic regions revealed that both QTLs and genes played important roles in rapeseed growth habits. Based on this study, we have provided very valuable data and resources for further rapeseed research.

## Author contributions

NW and XW organized and supported the entire study. NW, BC, KX, GG, FL, JQ, GY, JL, and HL performed the flowering time evaluation and SNP genotyping. NW also wrote and edited this manuscript.

### Conflict of interest statement

The authors declare that the research was conducted in the absence of any commercial or financial relationships that could be construed as a potential conflict of interest. The reviewer (NE) and handling Editor declared their shared affiliation, and the handling Editor states that the process nevertheless met the standards of a fair and objective review.
